# A novel method for optic disc localization using fast circlet transform and Chan-Vese segmentation

**DOI:** 10.1038/s41598-025-11257-7

**Published:** 2025-08-26

**Authors:** S. Gowthaman, Abhishek Das

**Affiliations:** Department of Mathematics, School of Advanced Sciences, Vellore Institute of Technology, Vellore, Tamil Nadu 632014 India

**Keywords:** Optic Disc Localization, Chan-Vese Segmentation, Fast Circlet Transform, Unsupervised Method, Minkowski Weighted K-Means, PDE-based Inpainting, Image processing, Machine learning, Vision disorders, Applied mathematics

## Abstract

Accurate localization and segmentation of the optic disc (OD) are considered crucial for the early detection of ophthalmic diseases such as glaucoma and diabetic retinopathy. Challenges such as image quality variability, high background noise, and insufficient edge information are often encountered by existing methods. To address these issues, an adaptive framework is proposed in which Fast Circlet Transformation (FCT) is combined with entropy-based features derived from retinal blood vessels for robust OD localization. Minkowski weighted K-means clustering is utilized to dynamically assess feature importance, thereby enhancing resilience to dataset variations. Following localization, partial differential equation-based image inpainting is employed for blood vessel removal, and OD segmentation is refined using the Chan-Vese active contour model. The method’s localization efficacy is demonstrated through extensive evaluations across multiple public datasets (DRISHTI-GS, DRIONS-DB, IDRID, and ORIGA), and segmentation performance metrics, including Dice coefficients of 0.94–0.95 and Jaccard indices of 0.9, are achieved on the ORIGA and DRISHTI-GS datasets. Through these results, the robustness and generalizability of the proposed method for clinical applications in retinal image analysis are highlighted.

## Introduction

The timely detection and treatment of retinal disorders, including glaucoma, diabetic retinopathy, hypertensive retinopathy, and age-related macular degeneration, are considered crucial for the prevention of irreversible vision loss and associated complications. Among these conditions, glaucoma is exemplified as a case demonstrating the urgent need for early intervention, as it is characterized by progressive optic nerve damage due to elevated intraocular pressure. It has been indicated by current research that global glaucoma cases will increase to 111.8 million by 2040. This issue is disproportionately observed in populations from Asia and Africa, where healthcare infrastructure and screening programs are often less accessible^1^. Consequently, the development of robust and accessible diagnostic tools has been identified as a critical priority in ophthalmology.

To address this pressing challenge, the Optic Disc (OD) is utilized as an important biomarker for identifying the severity of these diseases. Anatomically known as the Optic Nerve Head (ONH), the OD is represented as a circular, typically yellowish-white region in fundus images, characterized by the highest luminance intensity. It is marked as the convergence point of retinal vasculature and is identified as the exit site of retinal nerve fibers from the eye, thereby being recognized as a fundamental structure for clinical assessment.

Despite the OD’s distinctive morphological characteristics, including its circular shape, elevated brightness, and vascular convergence, substantial challenges are encountered by numerous image localization and segmentation methodologies in accurately delineating its boundaries and position. Many traditional localization and segmentation techniques^2,3^ have been focused on detecting the circular nature of the OD; however, this approach becomes problematic when boundary-depleted images, in which OD margins are poorly defined, are encountered.

Prior to the advent of deep learning, various supervised and unsupervised algorithms were proposed^4,5^. While unsupervised methods are shown to be robust against bright lesions, they are affected by vessel occlusion and imaging artifacts such as haze, blurry boundaries, and illumination variations. Although greater robustness to such distractions is demonstrated by traditional supervised learning methods, shallow learning approaches have been dependent on manual feature extractor design, which has relied heavily on expert experience and often has resulted in model overfitting^6^.

Despite significant progress made by deep learning approaches^7,8^ in OD detection, the requirement for extensive amounts of data has been noted as a limitation for robust classification, and the determination of optimal network hyperparameters remains a challenge. Given the significant variation in fundus images across geographical populations, the need for unsupervised domain adaptation^9^ has been emphasized to ensure consistent performance across diverse datasets.

To circumvent these limitations, a novel technique is proposed, inspired by the Fast Circlet Transform (FCT)^10^ and entropy-based features for OD localization^11^. Although FCT has previously been applied successfully to OD detection^10,12^, performance degradation has been observed in challenging clinical scenarios. To enhance performance, FCT is integrated with entropy features, which are known to provide crucial information about OD vascular convergence patterns.

Optimal OD localization is achieved in our approach by targeting image patches with strong circular patterns (indicated by high FCT coefficients) and significant blood vessel convergence (characterized by high entropy values). A weighted linear combination of these features across image patches is computed, with weights being adaptively learned using Minkowski weighted K-means clustering. This enables the system to be dynamically adjusted to diverse image conditions, thereby providing a data-driven strategy that improves robustness over fixed-weight methods. Following OD center localization, an active contour-based segmentation strategy is implemented. First, pre-processing is conducted using a Partial Differential Equation (PDE) based inpainting method to reduce vascular interference, followed by the application of Chan-Vese active contour segmentation initialized at the detected center. This hybrid methodology effectively is constructed by combining circlet features, vascular information, adaptive learning, and variational segmentation techniques for precise OD delineation.

The key contribution of this work is demonstrated through the development of a novel feature extraction and unsupervised adaptive localization method that addresses the practical limitations of deep learning approaches. Unlike deep models that require large annotated datasets and high-end GPUs for training, the proposed method is shown to be robust across different datasets due to its adaptive feature weighting and does not require retraining for new data. Most importantly, it is considered interpretable, offering clinically meaningful features such as circular structure strength and vessel convergence that can be easily understood by clinicians. This makes the proposed approach highly suitable for clinical use.

The literature is organized as follows: “Related Works” presents a review of existing methods in the field. “Methodology” introduces the proposed unsupervised framework. “Datasets and evaluation metrics” outlines the datasets and evaluation criteria employed in the study. “Results” presents the performance outcomes, while “Discussion” discusses comparative results and highlights limitations. Finally, “Conclusion and future scope” summarizes the key contributions and offers future research directions.

## Related works

OD feature extraction methodologies are generally categorized into appearance-based methods, model-based methods, and pixel-based approaches^13^.

In appearance-based methods, color, intensity, and texture properties are leveraged for OD detection. Automated OD detection was pioneered by Sinthanayothin et al.^2^, in which RGB fundus images were converted to HSI color space and regions of maximum intensity variance were identified. Subsequently, robustness was improved by Blanco et al.^3^ through the use of the Fuzzy Circular Hough Transform (FCHT), where fuzzy logic was employed to handle imperfect circular OD boundaries. The FCT was proposed by Chauris et al.^10^ to detect the OD efficiently by splitting images into circular components. Traditional circle detection algorithms such as the Hough Transform (HT) were outperformed through frequency-based circle analysis. However, these methods are found to struggle when the OD’s circular shape and color characteristics are distorted in pathological cases.

In model-based methods, prior geometric knowledge of OD morphology is incorporated, particularly by exploiting vessel convergence information. To account for anatomical variability, blood vessel components are extracted using various algorithms^14–21^, and the vessel convergence region is identified for OD detection. Ultrafast OD localization was achieved by Mahfouz and Fahmy^22^, in which vessel orientation was projected onto axes. Vessel density and directional features were combined with the HT by Zhang and Zhao^23^. The approach was further refined by Panda et al.^24^ through the exploitation of global and local vessel symmetry patterns. Although these methods are considered efficient, they remain sensitive to vascular abnormalities. When retinal blood vessels are thinned or are absent, accurate OD localization may not be achieved.

Hybrid approaches, where multiple features are integrated, have gained considerable attention in recent years. Morphological operations were fused with the Circular Hough Transform (CHT) by Aquino et al.^25^, while line operators and level sets were combined for boundary detection by Ren et al.^26^. Confidence scores based on intensity and directional vessel features were introduced by Xiong and Li^27^, resulting in improved robustness in pathological images. Despite such advancements, hybrid methods are observed to struggle in the presence of low-contrast OD regions and retinal abnormalities.

OD analysis capabilities have been fundamentally transformed by the deep learning revolution, where pixel-based approaches are employed for precise segmentation and localization at the pixel level. DRNet was developed by Hasan et al.^28^, in which Gaussian heatmaps were utilized for OD localization. Fuzzy clustering was integrated with active contours for segmentation by Abdullah et al.^4^. Transformer-based architectures such as JOINEDTrans^5^ and R-CNN frameworks^7^ shown to generalize effectively across diverse datasets. However, large annotated datasets are required, and domain adaptation challenges are encountered in clinical settings^8^.

Recently, unsupervised and lightweight methods were introduced to bridge the gap between accuracy and practical usability. Polar transforms were employed for rapid localization by Zahoor and Fraz^29^, while ConvNet region proposals were combined with mathematical priors by Dinc and Kaya^30^. The promise of unsupervised OD segmentation was highlighted by emerging foundation models such as the Fundus Segment Anything Model (FundusSAM), offering greater flexibility across datasets^31^. Recent advancements in attention modules and multi-scale fusion have been utilized to enhance OD feature extraction. While these approaches deliver strong performance, their dependence on annotated data remains, and reduced generalizability is often observed across diverse domains^32,33^. Additionally, fine boundary delineation and domain-specific variations are still found to be challenging.

Despite the considerable progress made across all methodological categories, existing approaches continue to face persistent challenges: appearance-based methods fail under pathological variation; model-based techniques struggle with abnormal vasculature; deep learning-based models require large datasets and lack adaptability; and current unsupervised methods trade off accuracy for computational simplicity. This gap has motivated the development of our adaptive approach, in which the geometric robustness of the FCT is combined with entropy-based vascular information through dynamic feature weighting, thereby enabling both precision and practical applicability without extensive training requirements.

## Methodology

The proposed method is designed to integrate frequency-domain coefficients from the FCT with spatial-domain vessel entropy features, allowing the structural and anatomical characteristics of the OD to be captured. The methodology is divided into five key stages: image preprocessing based on color dominance analysis, patch-based FCT for circular structure detection, blood vessel entropy analysis for anatomical feature extraction, adaptive feature fusion for robust OD localization, and a segmentation pipeline.

### Image preprocessing based on color dominance

Retinal fundus images exhibit significant variations in illumination, contrast, and color characteristics due to different acquisition systems, patient demographics, and pathological conditions. These variations can severely impact the performance of OD detection algorithms, as the disc’s circular boundary and vascular patterns may become obscured or distorted. Traditional preprocessing approaches using fixed color channels often fail to adapt to these diverse imaging conditions, leading to suboptimal feature extraction. In^34^, a preprocessing pipeline is proposed that addresses these challenges by automatically selecting the optimal color channels based on the specific characteristics of each image. This approach ensures consistent enhancement of crucial retinal structures across diverse datasets.

Following this approach, the input cropped fundus image $(512 \times 512)$, denoted as $I_{\text {RGB}}$, is considered in the RGB color space. The variance of the blue channel *B*(*i*, *j*) is then computed to determine the image’s color dominance:

$$\begin{aligned} \sigma ^2 = \frac{1}{mn} \sum _{i=0}^{m-1} \sum _{j=0}^{n-1} \left[ B(i, j) - \mu _B \right] ^2, \end{aligned}$$

where $\mu _B$ is the mean intensity of the blue channel, and $m \times n$ denotes the dimensions of the image. A low variance in the blue channel indicates red dominance (commonly observed in OD regions), while high variance suggests the presence of more chromatic variation, possibly due to pathology (e.g., exudates, hemorrhages). Using a threshold $\theta = 1500$, if $\sigma ^2 \le \theta$, the image is classified as red-dominant and the $a^*$ channel (red-green) in Lab space is enhanced; otherwise, the $b^*$ channel (blue-yellow) is enhanced^34^.

Subsequently, $I_{\text {RGB}}$ is converted to L*a*b* space, and Contrast Limited Adaptive Histogram Equalization (CLAHE) is applied to the selected $a^*$ or $b^*$ channel. The enhanced L*a*b* image is then converted back to RGB space. The enhanced RGB image is split, and CLAHE is applied to the green channel *G*(*i*, *j*) due to its superior vessel visibility and reduced noise susceptibility. The green channel closely resembles the lightness component and exhibits high contrast for vessels and OD regions. To remove noise while preserving edges, a bilateral filter is applied.

**Fig. 1:**
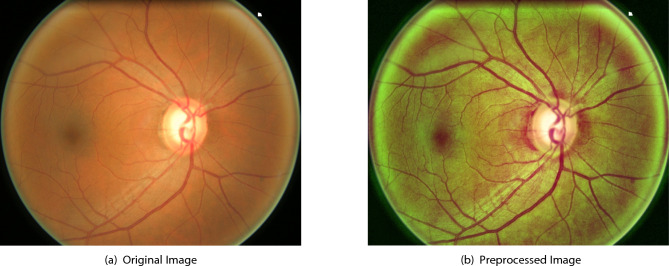
Comparison between original and preprocessed images.

The bilateral filter smooths flat regions while retaining edge features such as vessel boundaries and OD edges. Finally, brightness and contrast are automatically optimized using a linear transformation,

$$I_{\text {enhanced}} = \alpha \cdot I_{\text {filtered}} + \beta ,$$

where $\alpha = \frac{255}{\text {Gray}_{\max } - \text {Gray}_{\min }}, \quad \beta = -\text {Gray}_{\min } \cdot \alpha$. This scaling ensures dynamic range expansion and standardized intensity distribution, thereby enhancing the visibility of anatomical structures before entropy and circlet-based analysis. Figure 1 illustrates the preprocessing effect, where retinal blood vessels and OD pixel intensities are visibly enhanced, facilitating more effective feature detection.

### Patch-based FCT for circular structure detection

The FCT is a specialized algorithm for circular structure detection introduced by Chauris et al.^10^. Unlike traditional approaches such as the Hough Transform (HT), the FCT decomposes images into circular components across various radii and frequency scales while accounting for the finite frequency content of features. This enables efficient detection of imperfect circular shapes in noisy environments, making it particularly valuable for applications in medical imaging, astronomy, and oceanography^10,12,35,36^.

The FCT methodology is based on frequency-domain filter design. The process begins with the construction of one-dimensional frequency-domain filters $H_j(\xi )$, which partition the frequency domain to isolate bands associated with circular structures:

$$\begin{aligned} \sum _{j} |H_j(\xi )|^2 = 1, \quad H_j(\xi ) = {\left\{ \begin{array}{ll} \cos (\xi \pm \xi _j) & \text {if } |\xi \pm \xi _j| \le \frac{\pi }{N-1}, \\ 0 & \text {otherwise}, \end{array}\right. } \end{aligned}$$

where $j$ represents the scale index, $N$ denotes the number of filters, and $\xi _j = \frac{j - 1}{N - 1}$. The partition of unity property ensures complete coverage of the frequency domain without amplification or attenuation of any frequency component. These 1-D filters are then extended to two dimensions through a phase shift operation:

$$\begin{aligned} K_j(\xi _1, \xi _2) = e^{i|\xi |r_m} \cdot H_j(|\xi |), \end{aligned}$$

where $\xi = (\xi _1, \xi _2)$, $|\xi | = \sqrt{\xi _1^2 + \xi _2^2}$, and $r_m$ represents the radius of the target circular structure. These 2-D filters are designed to satisfy perfect reconstruction:

$$\begin{aligned} \sum _{j} |K_j(\xi _1, \xi _2)|^2 = 1. \end{aligned}$$

Subsequently, the preprocessed input image $I_{\text {enhanced}}$ is converted to grayscale $g(x, y)$. Then, the 2D Fast Fourier Transform (FFT) is applied to $g(x, y)$ to obtain its frequency representation:

$$\begin{aligned} \hat{g}(\xi _1, \xi _2) = \text {FFT}(g(x, y)). \end{aligned}$$

For each combination of radius $r_m$ and scale $j$, the transformed image is filtered:

$$\begin{aligned} \hat{c}_n(\xi _1, \xi _2) = \hat{g}(\xi _1, \xi _2) \cdot K_j(\xi _1, \xi _2), \end{aligned}$$

where $n = (r_m, x_p, j)$ encodes the center position, radius, and scale. The inverse FFT is then applied to obtain the circlet coefficients:

$$\begin{aligned} c_n(x, y) = \text {FFT}^{-1}(\hat{c}_n(\xi _1, \xi _2)). \end{aligned}$$

FCT-based approaches typically identify the OD location by selecting the pixel with the maximum circlet coefficient across the entire image. However, this global approach often fails when the OD region exhibits low contrast or is partially obscured, as pathological conditions tend to distort the circular appearance of the OD.

This issue is addressed by dividing the FCT coefficients into patches and analyzing each independently. In the proposed method, local contrast differences are exploited, reducing the impact of global illumination variations while enabling the detection of subtle circular features that may be missed in a global analysis. Additionally, patch-level entropy features are incorporated. In contrast to previous works that use radii ranging from 30 to 80 pixels (targeting the OD itself), this method focuses on the optic cup using smaller radii of $r = 10$ and $r = 20$ pixels. This choice is anatomically justified, as the optic cup represents the central depression within the OD and typically comprises one-third to one-half of the OD diameter.

The analysis is further constrained to scale $j = 2$ based on experimental results indicating that lower scales ($j = 1$) capture excessive high-frequency noise, while higher scales ($j \ge 3$) yield minimal additional information at the expense of increased computational cost.

This parameter selection optimizes the trade-off between detection accuracy and computational efficiency. The patch-based FCT implementation divides the coefficient map $c_n(x, y)$ into 100 non-overlapping patches $P_i$, each measuring $60 \times 60$ pixels. The patch size is chosen to ensure that even the smallest ODs in standard fundus images are wholly contained within at least one patch. For each patch, the maximum circlet coefficient from both radii at scale $j = 2$ is extracted:

$$\begin{aligned} \text {Max}\_\text {Coeff}_i = \max _{(x, y) \in P_i} \left( |c_{10,2}(x, y)|, |c_{20,2}(x, y)| \right) , \end{aligned}$$

where $i = 1, 2, \dots , 100$ indexes the patches. This approach enables the detection of circular structures even when they are located at patch boundaries or exhibit size variations.

The complete feature set from all patches and both radii is defined as:

$$\begin{aligned} \text {Feature Set}_1 = \left\{ \text {Max}\_\text {Coeff}_i^{(10)}, \text {Max}\_\text {Coeff}_i^{(20)} \mid i = 1, \ldots , 100 \right\} , \end{aligned}$$

where the superscripts denote the radius values.

### Blood vessel entropy analysis for anatomical feature extraction

To enhance detection accuracy across diverse imaging conditions, complementary entropy features are incorporated alongside FCT features. These entropy features are derived from retinal blood vessel patterns, which represent a key anatomical characteristic that remains relatively consistent even under challenging imaging conditions. The convergence of major retinal blood vessels at the OD creates a distinctive vascular pattern that can be leveraged for localization purposes. This anatomical arrangement persists even when the circular shape of the OD is distorted by pathology or when image contrast deteriorates. By analyzing the entropy of vessel distributions across image patches, regions exhibiting high vascular complexity–indicative of the OD region can be effectively identified.

A comprehensive approach to vessel segmentation is adopted, extending beyond conventional green-channel analysis. The methodology proposed by Coye^37^ is utilized, which leverages the complete color information via a weighted L*a*b* color model processed through Principal Component Analysis (PCA). The resulting binary image *B* contains segmented vessels (value = 1) against the background (value = 0), capturing the retinal vascular network with high fidelity.

To maintain consistency with the FCT analysis framework, the binary vessel image *B* is divided into the same set of 100 non-overlapping patches $P_i$, each of size $60 \times 60$ pixels. For each patch, entropy $H_i$ is calculated to quantify the randomness and complexity of the vessel distribution:

$$\begin{aligned} H_i = -\sum _{k \in \{0,1\}} p_k \log _2(p_k), \end{aligned}$$

where $p_0$ represents the proportion of background pixels (value = 0) and $p_1$ represents the proportion of vessel pixels (value = 1) in patch $P_i$.

Entropy serves as an effective measure of vessel pattern complexity and exhibits several key properties relevant to OD localization. High entropy values ($H_i \approx 1$) indicate patches with nearly equal proportions of vessel and background pixels, typically observed at the OD boundary where vessels enter and branch. Medium entropy values occur in regions with moderate vessel density, such as areas adjacent to the OD. Low entropy values correspond to patches dominated by either vessels or background, as commonly seen in peripheral retinal regions.

The complete vessel-based feature set is defined as:

$$\begin{aligned} \text {Feature Set}_2 = \{ H_i \mid i = 1, \ldots , 100 \}. \end{aligned}$$

At each patch, the availability of vessel information is represented through the entropy value, which complements the FCT-derived circularity feature. This synergy significantly improves the identification of OD candidates. A weighted linear combination of these two feature sets is then computed, and the patch with the maximum combined response is selected as the localized OD center.

### Adaptive feature fusion for robust OD localization

The complete feature set *Y* is constructed by concatenating the FCT-derived features with the vessel entropy features:

$$\begin{aligned} Y = \{\text {Feature Set}_1, \text {Feature Set}_2\} = \{\text {Max}\_\text {Coeff}_i^{(10)}, \text {Max}\_\text {Coeff}_i^{(20)}, H_i \mid i = 1, \ldots , 100\}. \end{aligned}$$

This unified feature space captures both structural (circular) and anatomical (vascular) characteristics of the OD region. The optimal patch containing the OD is identified by evaluating a weighted linear combination of the extracted features:

1 $$\begin{aligned} L_i = w_1 \cdot \text {Max}\_\text {Coeff}_i^{(10)} + w_2 \cdot \text {Max}\_\text {Coeff}_i^{(20)} + w_3 \cdot H_i, \end{aligned}$$

where $w_1$, $w_2$, and $w_3$ denote the weights associated with each feature. Initially, equal weights ($w_1 = w_2 = w_3 = \frac{1}{3}$) are employed, yielding promising results across several test cases. To further improve robustness under varying imaging conditions, a Minkowski weighted K-means clustering algorithm is implemented. This algorithm adaptively learns the optimal weights based on the specific characteristics of each image, enabling dynamic feature weighting.

By leveraging the complementary strengths of FCT based circular structure detection and entropy-based vascular analysis, the proposed adaptive fusion strategy ensures enhanced OD localization even in the presence of image noise, illumination variability, or pathological deformation.

The patch corresponding to the maximum linear combination score is selected as the final OD location:

2 $$\begin{aligned} \text {Patch}_{\text {max}} = \underset{i}{\text {argmax}} \{L_i \mid i = 1, \ldots , 100\}. \end{aligned}$$

#### Adaptive feature weighting

To determine suitable weights for the features, the Minkowski weighted K-means algorithm is adapted, which classifies the feature space based on the available information in each patch. As an unsupervised method, it does not require labeled data and effectively exploits the structure present in unlabelled datasets^38–41^.

The algorithm minimizes the following objective function:

$$\begin{aligned} F(S, C, w) = \sum _{k=1}^{\text {K}} \sum _{i=1}^{n} \sum _{v=1}^{M} s_{ik} w_v^\gamma \left| z_{iv} - c_{kv} \right| ^\gamma , \end{aligned}$$

where $\text {K} = 3$ is the number of clusters, $n = 100$ is the number of patches, $M = 3$ is the number of features, $s_{ik}$ indicates the assignment of patch *i* to cluster *k* (1 if assigned, 0 otherwise), $z_{iv}$ denotes the value of feature *v* for patch *i*, $c_{kv}$ is the centroid of cluster *k* for feature *v*, $w_v$ is the weight for feature *v*, and $\gamma$ is the Minkowski exponent adaptively selected via silhouette analysis.

To ensure stable clustering, an anomalous cluster initialization strategy is employed. A reference point is computed as the mean of all patch features, and the patch farthest from this reference is identified. Centroids are initialized by iteratively selecting points from the largest clusters, enhancing convergence stability.

The algorithm proceeds through three main iterative steps:

*Cluster Assignment:* Each patch is assigned to the nearest centroid using the weighted Minkowski distance:

$$\begin{aligned} d(z_i, c_k) = \sum _{v=1}^{M} w_v^\gamma |z_{iv} - c_{kv}|^\gamma . \end{aligned}$$

*Centroid Update:* Cluster centroids are recalculated as the mean of features from assigned patches:

$$\begin{aligned} c_{kv} = \frac{\sum _{i=1}^{n} s_{ik} z_{iv}}{\sum _{i=1}^{n} s_{ik}}. \end{aligned}$$

*Weight Optimization:* Feature weights are updated to minimize the objective function:

$$\begin{aligned} w_v = \frac{1}{\sum _{u=1}^{M} \left( \frac{E_v}{E_u} \right) ^{1/(\gamma - 1)}}, \end{aligned}$$

where $E_v$ represents the within-cluster dispersion for feature *v*.

Using the optimized weights, the weighted linear combination defined in Equation 1 is applied to each patch. The patch with the highest score, as defined in Equation 2, is selected as the candidate OD region. This patch, denoted as $\text {Patch}_{\text {max}}$, reflects the strongest combined evidence from both circular structure detection and vascular pattern complexity.

The proposed adaptive weighting scheme automatically emphasizes the most discriminative features while suppressing noisy ones, thereby enhancing robustness across diverse datasets without manual parameter tuning. In scenarios where FCT-derived features exhibit greater relevance than entropy features, the algorithm inherently assigns higher weights to the FCT terms, and vice versa.

Finally, the maximum-intensity pixel within the selected patch is identified, and its coordinates $(x_0, y_0)$ are taken as the localized OD center. This point is subsequently used to initialize the active contour model for precise OD segmentation.

### OD segmentation pipeline

Building upon the accurately localized OD center $(x_0, y_0)$ obtained from the hybrid FCT and entropy-based approach, a two-stage segmentation pipeline is implemented to address the challenges posed by retinal vasculature and pathological distortions. The process sequentially performs vessel removal and OD boundary segmentation as described below.

#### Vessel removal via PDE-based inpainting

In fundus images, blood vessels overlapping the OD boundary introduce intensity discontinuities, which adversely impact the performance of active contour-based segmentation techniques such as the Chan-Vese method. These discontinuities often cause contour leakage or convergence to incorrect boundaries. To mitigate this issue, PDE based inpainting approach is employed as a preprocessing step to selectively smooth vessel regions while preserving the global OD morphology^42^.

The original grayscale image is represented as $f(x, y): \Omega \rightarrow \mathbb {R}$, where $\Omega = [0, W] \times [0, H] \subset \mathbb {R}^2$, with *W* and *H* denoting the image width and height, respectively. A binary vessel mask $B(x, y): \Omega \rightarrow \{0, 1\}$ is used to identify vessel pixels in the image. The inpainting domain is defined as $D = \{ (x, y) \in \Omega \ | \ B(x, y) = 1 \}$, corresponding to pixels occupied by vessels requiring restoration. The complement $\Omega \setminus D$ defines the known, vessel-free regions.

The objective is to compute an inpainted image $u(x, y, t)$, which evolves over artificial time $t$ according to the heat equation with a data fidelity term:

$$\begin{aligned} \frac{\partial u}{\partial t} = \lambda \Delta u + \chi _{\Omega \setminus D}(f - u), \end{aligned}$$

where $\lambda$ controls the diffusion rate, $\Delta u$ is the Laplacian of $u$, and $\chi _{\Omega \setminus D}$ is the characteristic function defined as:

$$\begin{aligned} \chi _{\Omega \setminus D}(x, y) = {\left\{ \begin{array}{ll} 1 & \text {if } (x, y) \in \Omega \setminus D, \quad \text {(known region)} \\ 0 & \text {if } (x, y) \in D, \quad \text {(vessel region to be inpainted)}. \end{array}\right. } \end{aligned}$$

In regions where $\chi = 1$, the evolution of $u$ is driven toward the original image $f$, thereby preserving the intensities in known areas. In contrast, within vessel regions ($\chi = 0$), the evolution is governed solely by diffusion, promoting smooth intensity propagation from neighboring pixels. The initial condition is given by $u(x, y, 0) = f(x, y)$, for all $(x, y) \in \Omega .$

To prevent artifacts near image boundaries, homogeneous Neumann boundary conditions are imposed:

$$\begin{aligned} \frac{\partial u}{\partial x} \Big |_{x=0} = \frac{\partial u}{\partial x} \Big |_{x=W} = 0, \quad \frac{\partial u}{\partial y} \Big |_{y=0} = \frac{\partial u}{\partial y} \Big |_{y=H} = 0, \end{aligned}$$

where $W \times H$ denote the image width and height, respectively. These conditions ensure zero intensity flux across the boundaries.

The PDE is solved iteratively using explicit finite differences, and the iteration continues until convergence is reached, based on a predefined tolerance criterion $\Vert u^{n+1} - u^n\Vert < \varepsilon$. The resulting inpainted image, denoted as $I_w$, exhibits reduced vessel artifacts near the OD boundary, thereby facilitating robust segmentation using active contours.

**Fig. 2:**
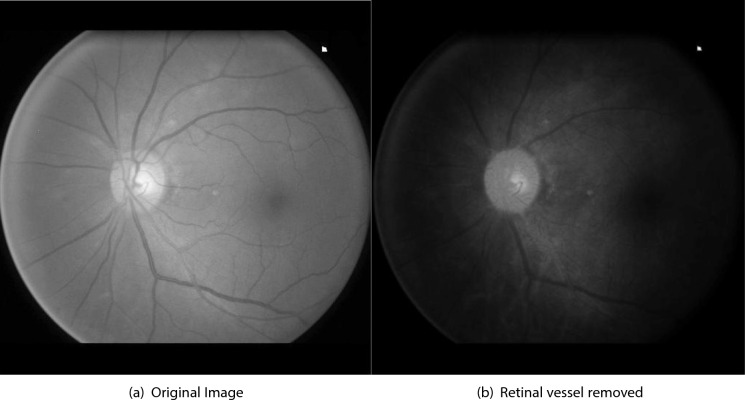
Vessel free fundus image.

In this approach, PDE-based inpainting is used as a preprocessing step to improve the performance and convergence of the Chan-Vese algorithm. Following inpainting, the green channel is discarded to suppress residual vessel artifacts. A weighted linear combination of the red and blue channels is computed to enhance the intensity transitions at the OD boundary. The enhanced image $I_w$ is defined as:

$$\begin{aligned} I_w = 0.7 \cdot I_R + 0.3 \cdot I_B, \end{aligned}$$

where $I_R$ and $I_B$ denote the red and blue channels, respectively, of the inpainted image. This vessel-suppressed image $I_w$ is used as input to the Chan-Vese segmentation method. An example of the resulting vessel-free fundus image is illustrated in Fig. 2.

#### Boundary segmentation via Chan-Vese model

Following vessel removal, the Chan-Vese segmentation method is applied to accurately delineate the OD region from the vessel-free image $I_w(x, y)$. This active contour technique formulates segmentation as a piecewise constant approximation problem and evolves a contour $C$ that separates regions of homogeneous intensity by minimizing an energy functional^43^.

The Chan-Vese energy functional is given by:

$$\begin{aligned} F(\alpha _1, \alpha _2, C) = \mu \cdot \text {Length}(C) + \lambda _1 \int _{\text {inside}(C)} |I_w(x, y) - \alpha _1|^2 \, dx \, dy + \lambda _2 \int _{\text {outside}(C)} |I_w(x, y) - \alpha _2|^2 \, dx \, dy, \end{aligned}$$

where $\alpha _1$ and $\alpha _2$ denote the average intensities inside and outside the contour $C$, respectively. The parameter $\mu$ controls the smoothness of the contour, while $\lambda _1$ and $\lambda _2$ govern the data fidelity terms inside and outside the contour.

To facilitate numerical implementation, the contour $C$ is represented implicitly as the zero level set of a Lipschitz-continuous function $\varphi$, such that $C = \{ (x,y) \in \Omega : \varphi (x,y) = 0 \}$. The segmentation functional is reformulated in terms of $\varphi$ using a regularized Heaviside function $H_\epsilon (\varphi )$ and its derivative, the Dirac delta function $\delta _\epsilon (\varphi )$:

$$\begin{aligned} \arg \min _{\alpha _1, \alpha _2, \varphi } \; \mu \int _{\Omega } \delta _\epsilon (\varphi ) |\nabla \varphi | \, dx \, dy + \lambda _1 \int _{\Omega } |I_w - \alpha _1|^2 H_\epsilon (\varphi ) \, dx \, dy + \lambda _2 \int _{\Omega } |I_w - \alpha _2|^2 (1 - H_\epsilon (\varphi )) \, dx \, dy. \end{aligned}$$

The regularized Heaviside function is defined as:

$$\begin{aligned} H_\epsilon (t) = \frac{1}{2} \left( 1 + \frac{2}{\pi } \arctan \left( \frac{t}{\epsilon }\right) \right) , \end{aligned}$$

and the corresponding regularized Dirac delta function is given by:

$$\begin{aligned} \delta _\epsilon (t) = \frac{1}{\epsilon \pi (1 + (t/\epsilon )^2)}. \end{aligned}$$

For a fixed $\varphi$, the optimal values of $\alpha _1$ and $\alpha _2$ are computed as the mean intensities within the regions defined by $H_\epsilon (\varphi )$. Subsequently, the level set function $\varphi$ is evolved using gradient descent as follows:

$$\begin{aligned} \frac{\partial \varphi }{\partial t} = \delta _\epsilon (\varphi ) \left[ \mu \operatorname {div}\left( \frac{\nabla \varphi }{|\nabla \varphi |}\right) - \lambda _1 \left( I_w - \alpha _1\right) ^2 + \lambda _2 \left( I_w - \alpha _2\right) ^2 \right] . \end{aligned}$$

This iterative update continues until convergence is achieved, resulting in a stable contour that accurately segments the OD region by balancing the contour’s smoothness with the fidelity to image intensities.

The parameters $\mu , \lambda _1, \lambda _2$, and $\epsilon$ are empirically selected based on the image quality and resolution. Upon convergence, the largest segmented circular region is extracted and designated as the final OD mask.

## Datasets and evaluation metrics

The effectiveness and generalizability of the proposed OD localization and segmentation framework are validated using four publicly available retinal fundus image datasets: DRISHTI-GS, DRIONS-DB, IDRID, and ORIGA. These datasets are selected to ensure diversity in image characteristics, particularly variations in OD contrast and structure, which are critical for evaluating the robustness of segmentation algorithms.

The DRISHTI-GS dataset consists of 101 high-resolution fundus images with well-defined OD boundaries. These images provide an ideal setting for performance evaluation under favorable contrast conditions. Similarly, the DRIONS-DB dataset contains 110 images where OD regions are clearly delineated, facilitating precise contour detection.

The IDRID dataset presents a more challenging scenario due to the low contrast between the OD and surrounding retinal background. For this study, only the segmentation subset comprising 103 images with annotated OD masks is utilized. These images are employed specifically for the tasks of OD localization and segmentation.

The ORIGA dataset includes 650 images and introduces further complexity, such as variability in OD and cup sizes, inconsistent textures, and the presence of imaging artifacts. Additionally, the limited number of annotated segmentation samples increases the risk of overfitting, particularly when detecting subtle structural deformations associated with early-stage glaucoma.

Together, these datasets represent a comprehensive evaluation benchmark, spanning from high-contrast, well-structured images to challenging, noisy cases. This ensures a rigorous assessment of the proposed framework’s accuracy, robustness, and generalization across diverse retinal imaging conditions.

### Evaluation metrics

To quantitatively assess localization precision, the normalized Euclidean distance between the predicted and ground truth OD centers is computed as follows:

$$\begin{aligned} \text {Normalized Distance} = \frac{\sqrt{(x_2 - x_1)^2 + (y_2 - y_1)^2}}{\sqrt{W^2 + H^2}}, \end{aligned}$$

where $(x_1, y_1)$ and $(x_2, y_2)$ denote the coordinates of the ground truth and predicted OD centers, where *W* and *H* represents the width and height of the image as mentioned early. For segmentation accuracy evaluation, the Dice Coefficient and the Jaccard Index (Intersection over Union, IoU) are utilized. These metrics effectively quantify the spatial overlap between the predicted segmentation and the ground truth region.

- *Dice Coefficient:* The Dice score measures the similarity between two binary masks and is given by:
  $$\begin{aligned} \text {Dice} = \frac{2 \times |A \cap B|}{|A| + |B|}, \end{aligned}$$
  where $A$ is the predicted segmentation and $B$ is the ground truth. Dice score closer to 1 indicates a higher degree of overlap.
- *Jaccard Index (IoU):* The Jaccard Index evaluates the intersection over union of the predicted and ground truth regions and is defined as:
  $$\begin{aligned} \text {IoU} = \frac{|A \cap B|}{|A \cup B|}. \end{aligned}$$

These metrics are computed for each image in the dataset, and the mean values are reported to summarize the overall segmentation performance.

## Results

The performance of the proposed OD localization approach was evaluated on three publicly available datasets: IDRID, DRION, and DRISHTI-GS. The results, summarized in Table 1, highlight the comparative performance of the adaptive and non-adaptive methods using normalized distance metrics.

On the IDRID dataset, the adaptive method achieved a mean normalized distance error of 0.2493, compared to 0.2539 for the non-adaptive method, reflecting a consistent improvement of approximately 1.8%. The median error decreased from 0.2628 to 0.2520, and the minimum error improved from 0.0526 to 0.0479, indicating enhanced precision in optimal scenarios.

**Table 1:** Error Analysis Matrix for OD Localization (Normalized Distance Metrics).

Dataset	Method	Mean	Median	Std Dev	Max	Min
IDRID	Non-Adaptive	0.2539	0.2628	0.0902	0.4894	0.0526
	Adaptive	0.2493	0.2520	0.0911	0.4894	0.0479
DRION	Non-Adaptive	0.0745	0.0583	0.0824	0.6864	0.0031
	Adaptive	0.0736	0.0583	0.0816	0.6864	0.0031
DRISHTI-GS	Non-Adaptive	0.0756	0.0713	0.0423	0.1928	0.0062
	Adaptive	0.0771	0.0778	0.0431	0.1928	0.0062

On the DRION dataset, the adaptive method outperformed the non-adaptive version with a reduced mean error (0.0736 vs. 0.0745) and a lower standard deviation (0.0816 vs. 0.0824), suggesting more consistent localization–a desirable attribute for clinical applications.

In contrast, the DRISHTI-GS dataset presented a marginal anomaly. The non-adaptive method exhibited a slightly better mean error (0.0756 vs. 0.0771); however, the standard deviation remained comparable between the two methods. This result suggests that the performance difference is negligible and unlikely to be clinically significant.

Overall, the adaptive method consistently demonstrated improved or equivalent performance across datasets, particularly excelling in challenging imaging conditions as observed in the IDRID and DRION datasets. The observed reductions in mean error and variability establish the adaptive approach as a more robust and reliable solution for OD localization across diverse retinal fundus image datasets.

### OD segmentation performance

The proposed OD segmentation method was rigorously evaluated on two publicly available and clinically challenging datasets: ORIGA and DRISHTI-GS. These datasets were selected to assess the robustness and generalizability of the method across a wide range of image qualities, pathological conditions, and demographic variations.

Table 2 presents the quantitative performance metrics, namely Dice Coefficient and Intersection over Union (IoU), for the proposed method on each dataset.

On the ORIGA dataset, which comprises 650 images characterized by diverse pathological features including exudates and OD deformation, the method achieved a median Dice coefficient of 0.94 and an IoU of 0.90. These results demonstrate the method’s ability to maintain high segmentation accuracy even in the presence of noise, anatomical variability, and retinal pathologies.

On the DRISHTI-GS dataset, a slightly higher median Dice coefficient of 0.95 was observed, while the IoU remained consistent at 0.90. This marginal improvement is attributed to the dataset’s higher imaging quality and more standardized acquisition protocol, which facilitate more accurate boundary detection.

The consistently high Dice coefficients (above 0.94) across both datasets indicate excellent agreement between the predicted and ground truth OD boundaries. Similarly, the high IoU scores (0.90) confirm precise spatial overlap, affirming the method’s clinical applicability.

**Table 2:** Quantitative OD Segmentation Performance of the Proposed Method.

Dataset	Dice Coefficient	IoU
ORIGA	0.94	0.90
DRISHTI-GS	0.95	0.90

**Fig. 3:**
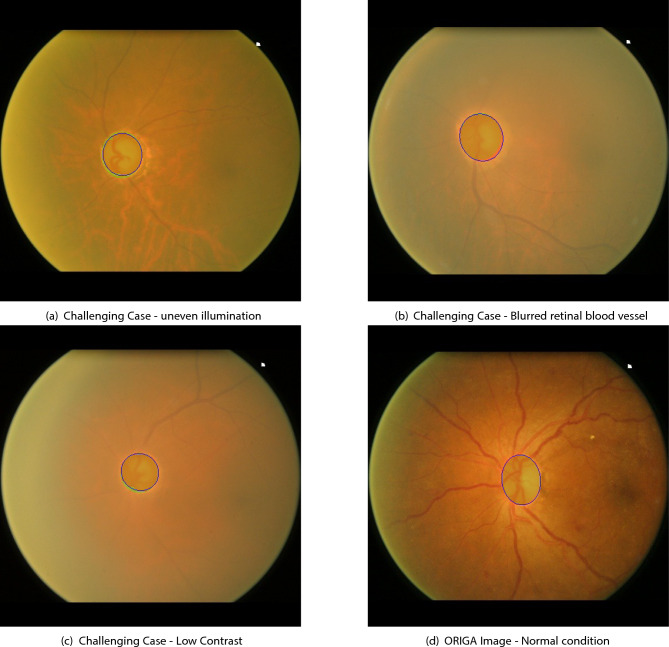
Qualitative segmentation results demonstrating method performance across diverse cases. Blue contours represent our segmentation output, while green regions indicate ground truth annotations.

Qualitative validation is provided in Fig. 3, which presents segmentation overlays where predicted contours (in blue) are compared against ground truth boundaries (in green). These visual results further validate the robustness of the proposed method, showing high boundary accuracy across both normal and pathological conditions. Notably, the method maintains segmentation reliability in challenging scenarios, including low-contrast fundus images and glaucomatous changes.

## Discussion

### Comparative analysis with state-of-the-art methods

To establish the clinical relevance and technical merit of the proposed segmentation framework, comprehensive comparisons were conducted with several state-of-the-art OD segmentation techniques using multiple evaluation metrics. Table 3 presents the Dice Coefficient and Jaccard Index (IoU) for various methods across standard datasets.

The proposed method achieved Dice scores of 0.95 and 0.94 on the DRISHTI-GS and ORIGA datasets, respectively, demonstrating competitive performance. While certain deep learning-based approaches, such as MSGANet-OD ^44^ and the modified U-Net with ResNet-34 ^45^, reported marginally higher Dice coefficients (0.96), the proposed method maintains clinical viability without the reliance on large-scale annotated data or extensive training.

**Table 3:** Comprehensive Comparison of OD Segmentation Methods.

Study	Method	Dataset	Dice Coefficient	Jaccard Index
^7^	RCNN	RIM-ONE DL	0.9024	0.9246
		DRISHTI-GS	0.9488	0.9574
		Combined	0.9431	0.9295
^46^	SAM	DRISHTI-GS	0.88	–
^44^	MSGANet-OD	DRISHTI-GS	0.96	0.913
^47^	BFO	DRISHTI-GS	0.95	–
^45^	Modified U-Net with ResNet-34	DRISHTI-GS	0.96	0.93
^8^	GWO + YOLOV7	DRISHTI-GS	0.9415	0.904
^48^	U-Net	ORIGA	0.962	–
		REFUGE	0.965	–
		RIM-ONE v3	0.910	–
		DRISHTI-GS	0.943	0.904
**Our Method**	**Proposed Approach**	**ORIGA**	**0.94**	**0.90**
		**DRISHTI-GS**	**0.95**	**0.90**

In addition, the proposed method was compared with frequency-domain based segmentation techniques. Table 4 outlines this comparison on the DRISHTI-GS dataset. While methods such as wavelet-enhanced CNN ^49^ and Fourier contour models ^50^ achieved higher Dice scores (up to 0.9765), they also imposed increased computational complexity and greater reliance on data-driven learning or shape priors. In contrast, the proposed method offers a balanced trade-off between accuracy and computational efficiency.

**Table 4:** Performance Comparison with Frequency-Domain Methods on DRISHTI-GS Dataset.

Method	Technique	Dice (DRISHTI-GS)	Remarks
**Proposed Method**	FCT + Entropy + Chan-Vese	**0.9500**	Balanced accuracy and efficiency
Sun et al. (2023) ^49^	Wavelet-enhanced CNN	0.9759	High accuracy; computationally intensive
Chen et al. (2025) ^50^	Fourier contour model	0.9765	Smooth boundaries; sensitive to shape priors

### Statistical validation and significance testing

To establish the statistical significance of the segmentation results, comparative analyses were performed against established methods using the DRISHTI-GS dataset. Specifically, statistical tests were conducted against U-Net and SAM ^46^, both of which are widely adopted for OD segmentation.

No statistically significant differences were observed when comparing the proposed method with U-Net ($p = 0.598$, Cohen’s $d = 0.117$) or SAM ($p = 0.609$, Cohen’s $d = 0.206$), though small positive effect sizes were noted in favor of the proposed approach. These outcomes suggest that, while differences are subtle, the method consistently achieves comparable or slightly superior results.

Appropriate statistical methodologies were employed based on the data distribution: paired t-tests for normally distributed data, and Wilcoxon signed-rank tests otherwise. The small effect sizes ($d < 0.3$) indicate consistency without significant variability across test cases.

Unlike deep learning approaches that often require extensive labeled datasets and are prone to overfitting, especially under limited data availability, the proposed method exhibits robust generalization capabilities. This resilience stems from its unsupervised design and the adaptive weighting mechanism in the localization stage. Theoretically, this adaptivity aligns with the principle of maximum likelihood estimation, wherein local feature weights are dynamically adjusted to reduce reliance on any single modality, thereby enhancing robustness across varying imaging conditions and pathological scenarios.

### Failure case analysis and limitations

**Fig. 4:**
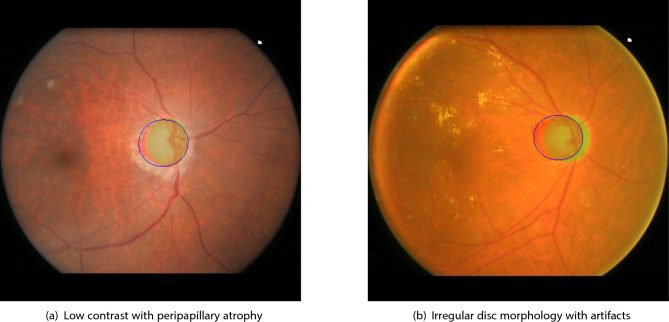
Representative failure cases demonstrating limitations of the proposed method. (**a**) Low contrast and peripapillary atrophy result in indistinct boundaries. (**b**) Irregular disc shape and image artifacts challenge contour-based segmentation algorithms.

Despite the overall strong performance, specific failure modes were identified, particularly within the ORIGA dataset, that merit detailed discussion. Figure 4 presents representative examples of segmentation failures, offering insights into scenarios where the proposed method encounters limitations. In certain cases, the OD boundary appears indistinct or blurred, reducing the efficacy of edge-preserving and gradient-based techniques. This often results in leakage of the segmentation contour into adjacent tissues. As illustrated in Fig. 4a, the presence of peripapillary atrophy around the OD combined with low image contrast, leads to ambiguous transitions between disc and background regions. This degrades the performance of intensity-driven models such as Chan-Vese. Figure 4b highlights a case where the OD exhibits non-circular, pathological morphology. Such irregularity challenges the assumptions of circularity or ellipticity embedded in many segmentation frameworks. Imaging artifacts, such as glare or blur, can further confound contour evolution, especially when they overlap with the OD boundary region.

These failure cases emphasize the intrinsic complexity of medical image segmentation and underscore the importance of developing robust preprocessing pipelines. Future work could explore integrating texture-based priors, shape-aware post-processing, and adaptive contrast enhancement to further mitigate such limitations. Additionally, hybrid models that combine data-driven learning with prior-based regularization may offer improved resilience to pathological variation and imaging noise.

## Conclusion and future scope

An enhanced method for OD localization is introduced in this research, utilizing features derived from the FCT and blood vessel-extracted images, in conjunction with the Chan-Vese active contour method. Substantial improvements over conventional image processing techniques are demonstrated, with performance levels comparable to deep learning models achieved without the reliance on annotated training data, particularly on challenging datasets such as ORIGA and DRISHTI-GS. In future work, the proposed method is intended to be incorporated as a preprocessing module within lightweight deep learning architectures. By leveraging the unsupervised nature and efficiency of the current framework, it is anticipated that both the robustness and accuracy of subsequent learning-based models will be enhanced across diverse retinal image datasets. Furthermore, to mitigate the influence of blurry boundaries often occurring in fundus images due to illumination variance and acquisition artifacts, advanced frequency-domain filtering strategies, such as adaptive thresholding in the FCT domain or multiscale vessel suppression, are planned to be explored. This strategic integration aims to endow deep learning systems with improved resilience and efficiency, thereby facilitating their deployment in resource-constrained clinical environments and large-scale population screening programs. Ultimately, the proposed direction underscores a commitment to reconciling precision with practicality in the field of medical image analysis.

## Data Availability

The datasets used in this study are publicly available at the following links: DRISTI-GS: https://www.kaggle.com/datasets/deathtrooper/multichannel-glaucoma-benchmark-dataset, DRIONS-DB: http://www.ia.uned.es/~ejcarmona/DRIONS-DB/BD/DRIONS-DB.rar, IDRID: https://www.kaggle.com/datasets/aaryapatel98/indian-diabetic-retinopathy-image-dataset, ORIGA: https://www.kaggle.com/datasets/arnavjain1/glaucoma-datasets. The implementation code for the proposed method is available at: https://github.com/Gowthaman803/Optic_Disc/tree/main
